# A Typical Case Report: Internet Gaming Disorder Psychotherapy Treatment in Private Practice

**DOI:** 10.3390/ijerph18042083

**Published:** 2021-02-21

**Authors:** Daryl Wayne Niedermoser, Andreas Hadjar, Vivien Ankli, Nina Schweinfurth, Claudia Zueger, Renanto Poespodihardjo, Sylvie Petitjean, Gerhard Wiesbeck, Marc Walter

**Affiliations:** 1 Department of Addictive Disorders, University Psychiatric Clinics (UPK), University of Basel, 4002 Basel, Switzerland; vivien.ankli@upk.ch (V.A.); nina.schweinfurth@upk.ch (N.S.); renanto.poespodihardjo@upk.ch (R.P.); sylvie.petitjean@upk.ch (S.P.); gerhard.wiesbeck@upk.ch (G.W.); marc.walter@upk.ch (M.W.); 2 Department of Economics, Kalaidos University of Applied Sciences, 8050 Zürich, Switzerland; 3 Institute of Education and Society, Maison des Sciences Humaines, University of Luxembourg, 4366 Esch-sur-Alzette, Luxembourg; andreas.hadjar@uni.lu; 4 Faculty of Philosophy and History, University of Basel, 4001 Basel, Switzerland; claudia.zueger@unibas.ch

**Keywords:** internet gaming disorder, psychotherapy, non-substance-related disorders, non-substance-use disorders, behavioral disorders

## Abstract

*Background*: Online or internet gaming disorder (IGD) is currently not recognized as a mental disorder in the actual Diagnostic and Statistical Manual of Mental Disorders (DSM-5), although it is an emerging disease. Non-substance-related addictions often have similarities with substance addictions. It is therefore important to have a good understanding of the client but also to have a good endurance. Due to the rise of e-sports, there is an anticipated and therefore possible trend to have many more patients with a non-substance addiction. There are many parallels, for instance tolerance, withdrawal and social problems, resulting from an increasing investment of time spent on the internet. *Case presentation*: To reduce possible inhibition in treating a patient with IGD, we present a case of a 19-year-old adolescent man who exhibited IGD and showed social problems associated with his addiction. *Conclusions*: This paper shows the importance and the effects of treating a non-substance addiction with cognitive behavioral therapy (CBT). After having successfully coped with an addiction, several shifts in addiction were often reported. In this case, no shifts were reported. The absence of such shifts makes our case a distinct and unique case. This is not a multimorbidity case, and that is the reason why we think this is an excellent example to show what we achieved, how we achieved it, and what we could establish. Of course, additional research and manuals are urgently needed.

## 1. Background

What is the difference between an intensive gamer and a professional e-sports player? Some people would assume that the difference lies in the notion of success (mostly money and fame). Time spent playing the game is probably an additional factor. But when is it excessive use or an addiction? Internet gaming disorder (IGD) is currently not recognized as a mental disorder in the actual Diagnostic and Statistical Manual of Mental Disorders (DSM-5), although it is an emerging disease. Due to the rise of e-sports, there is an anticipated trend to have more patients with a non-substance addiction.

Addictions are not easy to treat. It is often nerve-wracking for the client, but also for the therapist. This case report is based on the CARE checklist (see Figure S1). We present the case in private practice of a behavioral addiction to “online gaming”.

## 2. Case Description

Until recently, there was hardly any objective discussion beyond ideological and/or emotional tones. There were nine criteria proposed to diagnose IGD from the American Psychiatric Association (APA, 2013, 795ff) [1]. These criteria were based on preliminary research, which compared video game use to gambling addiction [2]. The following criteria are listed:

- Increasing amounts of time invested (tolerance)
- Escape of adverse moods (regulation)
- Loss of relationships
- Reduced participation in other activities (isolation)
- Deceit in order to be able to play
- Continuation of playing despite adverse consequences
- Difficulty reducing video game use
- Symptoms of withdrawal after discontinuation of video game use

According to the Diagnostic and Statistical Manual of Mental Disorders (DSM-5), patients with clinically relevant IGD should exhibit at least five or more of the above listed criteria during a twelve-month period. The clinical significant dysfunction results in severe social and emotional problems. Dan met all of the above listed nine criteria. He played on a daily basis about twelve hours a day. In addition, as is typical of many other addictions, his addictive gaming started with a little dose of “only a few minutes”. That intense gaming time in combination with almost no sleep resulted in poor performance. This is in line with findings of Mössle et al. (2010) that extensive media use and gaming negatively affect learning, as it may draw some attention away from learning and divert cognitive abilities away from school-related activities [3,4]. Moreover, the patient tried to conceal his gaming use at first, but then he continued, although his parents were confronting him with his possible gaming addiction.

The following case describes a 19-year-old Swiss male who acquired no psychiatric comorbidities before. Following, we use “Dan” as a pseudonym. Dan grew up in a typical Swiss family in a rural city in the Canton of Baselland (Switzerland). He had a three-year-older sister, but unfortunately, she died seven years before when Dan was twelve years old. Dan began playing video games starting at the age of five (see Figure S2, “Timeline”). His parents had two well-paid jobs and were living in a huge house. At the age of eight, he was officially allowed to play video games for one hour per day. However, he admits later that he often played for a longer time. In fact, he played much more because his parents were not home often. At the age of five, he started with learning games (sort numbers). At the age of eight, he quickly moved to social games, and by the age of eleven he turned to first-person shooter games. The whole family gave him all the games and consoles he wanted; they did not realize that his gaming behavior could be an expression of an addiction.

When Dan was about eight, he had friends to play with on PlayStation and on a personal computer (PC)—but only at his home. Soon, Dan realized in school that he was not performing very well in physical education (PE). He remembers being laughed at by the PE teacher and that he was getting an unsatisfactory grade. Maybe he was a little bit chubby, as he remembers it. According to him, this was a very negative and kind of traumatic experience for him, and so he focused more and more on his gaming skills.

At the age of eleven, he figured out the password for internet access (and child restriction) at home so he could play some first-person shooter games on a multiplayer-level (mainly old-school first-person shooter games such as Counter-Strike and Doom). He told me that his father used to play first-person shooter video games when his father was young. The negative influence of violent video games has been investigated as a neural correlate of social capabilities [5,6]. It has been shown that particularly violent games increase the risk of social withdrawal, whereby social support is a known protective factor against mental illness [6].

His social interaction was more and more limited to school only. Fortunately for him, he was an excellent pupil so that he did not need to invest much time in learning. Unfortunately, neither his parents nor the school realized what was happening for a long time.

At the age of sixteen, he began his apprenticeship in an office, but he soon began to run into troubles there. He was always tired and was late for work. The professional school grades were not as good as they were in school. Therefore, the company contacted his parents. Finally, they figured out that he was playing online games for about twelve hours daily; primarily first-person shooter games and, for “relaxation” (as he calls it), some role-playing games. This was the first time that his parents became aware of his excessive gaming. They always thought that their boy was just going through a rough phase (initially because of the death of their daughter).

Nevertheless, the problem did not stop there. His parents locked up his gaming stuff and hoped to stop the problem, but then Dan started to game on his smartphone at his workplace. Thus, his parents failed to prevent him from becoming addicted, as Dan only shifted from a stationed gaming device to a mobile device and did not reduce gaming. Dan subsequently lost his apprenticeship but was lucky to find another place. However, in order to keep the apprenticeship, the new company made a deal with Dan and his parents. He should find a psychotherapist. That is how he came to the psychiatric practice, and for the next eight months he came in regularly for psychotherapy. The sessions were mainly held once a week. At first, we had to establish some basic rules (being on time, keeping appointments, and so on) for the patient but also had to inform his parents and the new company about Dan’s progress. Dan’s parents and boss were informed to assure a transparent and constant flow of information for all involved people. Baseline assessment was measured using the Hamilton-21 (Hamilton), the Insomnia Severity Index (ISI), and average game time per day (AGT) (see Table 1). The Hamilton questionnaire is an experts’ rating scale for depression. The Insomnia Severity questionnaire measures a patient’s current perception in terms of severity, stress, and impairment during the last two weeks. AGT focuses on self-reported game time per day.

**Figure 1:**
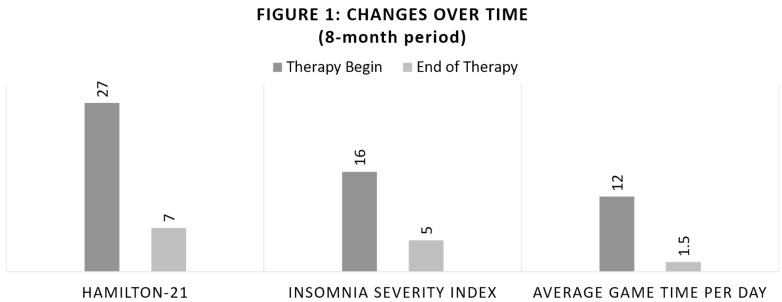
Changes over time (8-month period). Note: own depiction.

During the first few sessions, the patient had trouble understanding that his gaming behavior was related to an addiction problem. On the contrary, he rather anticipated the positive outcomes of gaming in terms of becoming a famous player and getting rich. In a next step, he accepted that he sometimes tried to quit gaming, but on the other hand, he interpreted his professional failure as a sign of fate in order to become a professional e-sports player. However, he quickly adapted his focus, and he agreed to reduce the time spent online (for gaming) (see Figure 1 and Table 1, below). As complementary psychoeducation, he was informed that there is often a shift to another addiction. The client was made aware of this, and it was often asked about at the beginning of a therapy session.

An important motivator for the therapy, and thus the patient’s motivation, was that he had done very poorly at a tournament. This made him realize that he maybe was “in a kind of bubble”, that he did not perceive the real situation adequately, and that his family may be right after all. His own treatment goals were to keep his apprenticeship and concurrently to reduce the hours spent online (gradually). We soon realized that he was not familiar with social interactions and showed signs of social anxiety.

Applying cognitive behavioral therapy (CBT) for eight months (psychoeducation, goal attainment scale) was quite successful. The patient was able to accept that he had a gaming addiction, and he reduced his game time down to about 90 min per day. During our therapy, the focus was on various methods commonly used in CBT, including psychoeducation (e.g., sleep and functioning, addiction, control, and desire); cognitive restructuring (e.g., evaluation of a break in play, the relevance and importance of offline vs. online relationships, individualized handling of craving, development of alternative actions); and mindfulness (e.g., What is good for me?). He was given the task of making himself knowledgeable on the Internet about his addiction problem. So he started doing some research to find out more. In therapy sessions we discussed what we had learned. A main focus was on where and what he had learned. We discussed what applied to him and, even more importantly, what did not. For him, this was a major reduction in game time and a huge success for him. Furthermore, his subjective sleep quality improved, and his social life became more important (see Table 1, above). The therapeutic process had accelerated itself through that. He became more socially engaged at a local church. In addition, he performed well at the final stages of his apprenticeship with an anticipated successful graduation in summer 2020.

While he had been developing new hobbies and fields of interest, including elements of social life, he was not interested in having an intimate relationship with another person. He realized that he was able to game without returning to his old addictive gaming patterns but that he also needed to reflect on this and to consciously focus on his obligations and health. During our treatment, the patient was checked for other signs of addiction. However, the results were negative, as none of the amounts exceeded the typical usage of a young adolescent man. Dan rarely consumed alcoholic beverages but quite a lot of energy drinks. He also rarely consulted pornographic material but not in an excessive way.

## 3. Discussion

This case is an example of the complexity of psychosocial factors that are included in pathologic gaming disorder. Parents and peers try to help, but their reaction rather reinforces the unwanted behavior. Dan began to play video games at an especially vulnerable young age during a main socialization phase. He was not a troublemaker in school and got good grades. Maybe the first signs had been overlooked (higher time spent playing games).

That starting age is also particularly vulnerable. Dan’s early addiction was disturbing his individual, social, and emotional development. Important drivers or triggers of his addiction were his parents and their parenting style, as well as the patient’s need for satisfaction or attention as a young adult. Most games “trick the player”, as the players perceive real satisfaction when they have completed a gaming level or a task. Chaput et al. (2011) showed that video games are associated with pleasure and excitement and that they stimulate the HPA axis, resulting in increased blood pressure and heart rate [7]. Violent video games produced a lower activation of limbic and temporal areas in the brain [6]. In several fMRI scans, the arousal can be shown in brains of video game addicts [8] as well as internet addicts [5]. A craving-related isolated brain pathway related in response to internet cue could not be identified [5]. As the fMRI scans showed only male video game addicts, IGD seems to affect a large number of males. The reasons may lie in the type and nature of the games programmed [9].

The impact of IGD on young children and possible significant problems later on in life needs further research. It seems that many discussions are focused on what the individual believes and not necessarily on empirical facts. However, in the present pretest–posttest study, the treatment outcome was measured after eight months of therapy.

Often other addictions are used as a substitute. For example, excessive buying, hoarding, or substance-related addictions such as alcohol or tobacco cigars. This case was not one of those. No shifts were reported. Repetitive psychoeducation about the possible shifts toward a substitute was very helpful in the therapeutic process. He was made aware of that and realized that he was not alone with his problem. He started doing some internet research to find out more. In therapy sessions, we discussed what we had learned, in particular what he had learned, what applied to him, and what did not. He was interested in understanding his addiction, and we routinely had psychoeducation on the therapy plan.

The same questionnaires were used (see Table 1) before and after therapy: the Hamilton-21, the Insomnia Severity Index, and the average game time per day. The results showed an improvement in every measured aspect. The Hamilton-Depression-Sumscore was reduced from 27 to 7 (from severe depression to no depression). The subjective sleep complaints, measured with the Insomnia Severity Index, decreased over time from 16 to 5 (from clinical insomnia with moderate severity to no clinically significant insomnia), and the average game time spent per day decreased as well from 12 to 1.5 h (from severe to moderate).

## 4. Conclusions

We conclude that this case might be of broader interest for experts and possibly for the family members of gaming addicts, as the severe consequences for the patient’s life were profoundly reduced in our example, while the patient changed his behavioral patterns from addictive behavior to a moderate behavior. Dan overcame his gaming disorder, but he is still attracted to gaming in a non-addictive way. With professional help and support, there is a realistic chance to overcome a gaming addiction. It doesn’t seem to be necessary to apply a zero tolerance approach and ask the patient to give up gaming entirely. Although the costs for frequent psychotherapy (once a week) seem to be initially high, the positive effects on health, family, and social life appear to massively outweigh the financial input. In this case, the patient could keep and probably successfully finish his apprenticeship. This is of major importance for his later prospects to live a self-determined and independent life.

From a retrospective perspective, it is hard to tell if Dan’s social anxiety was the result of social isolation and setting the priorities to video gaming, or if he initially began to play video games as an avoidance strategy in response to his isolated social situation. Our working hypothesis was a mixture of both, but the avoidance strategy could be used in combination with his negative experience with his PE teacher. After having successfully coped with an addiction, several shifts in addiction were often reported (e.g., pornography) [10]. This was discussed regularly. In this case no shifts were reported. The absence of such shifts makes our case a distinct and unique case.

## Figures and Tables

**Table 1:** Pre and post measurement.

	Beginning of Therapy	End of Therapy
Hamilton-21	Severe Depression	No Depression
Insomnia Severity Index	Clinical insomnia with moderate severity	No clinically significant insomnia
Average game time per day	Severe time spent	Moderate time spent

## Data Availability

All data underlying the results are available as part of the article and no additional source data are required.

## Supplementary Materials

The following are available online at https://www.mdpi.com/1660-4601/18/4/2083/s1, Figure S1: CARE Checklist: For the original draft of the case report, the CARE Checklist was used to structure it; Figure S2: Timeline.
