# Supplementary material for: A Typical Case Report: Internet Gaming Disorder Psychotherapy Treatment in Private Practice
Source: Int J Environ Res Public Health. 2021 Feb 21;18(4):2083. doi: 10.3390/ijerph18042083 (PMC7924621; doi:10.3390/ijerph18042083)
Supplement: Supplementary file 1 [file ijerph-18-02083-s001.pdf]

Supplementary Material

CARE case report guidelines — **CARE Checklist of information to include when writing a case report**

Topic	Item	Checklist item description	Reported on Line
Title	1	The diagnosis or intervention of primary focus followed by the words "case report"	Title
Key Words	2	2 to 5 key words that identify diagnoses or interventions in this case report, including "case report"	15
Abstract (no references)	3a	Introduction: What is unique about this case and what does it add to the scientific literature?	17
	3b	Main symptoms and/or important clinical findings	51+ff
	3c	The main diagnoses, therapeutic interventions, and outcomes	82+ff
	3d	Conclusion—What is the main "take-away" lesson(s) from this case?	140+ff
Introduction	4	One or two paragraphs summarizing why this case is unique (may include references)	21+ff
Patient Information	5a	De-identified patient specific information.	42+ff
	5b	Primary concerns and symptoms of the patient.	42+ff
	5c	Medical, family, and psycho-social history including relevant genetic information	57+ff
	5d	Relevant past interventions with outcomes	none
Clinical Findings	6	Describe significant physical examination (PE) and important clinical findings.	none
Timeline	7	Historical and current information from this episode of care organized as a timeline	42+ff
Diagnostic Assessment	8a	Diagnostic testing (such as PE, laboratory testing, imaging, surveys).	
	8b	Diagnostic challenges (such as access to testing, financial, or cultural)	77+f
	8c	Diagnosis (including other diagnoses considered)	77+ff
	8d	Prognosis (such as staging in oncology) where applicable	82+ff
Therapeutic Intervention	9a	Types of therapeutic intervention (such as pharmacologic, surgical, preventive, self-care)	29+ff
	9b	Administration of therapeutic intervention (such as dosage, strength, duration)	93+ff
	9c	Changes in therapeutic intervention (with rationale)	93+ff
Follow-up and Outcomes	10a	Clinician and patient-assessed outcomes (if available)	see table and grafic
	10b	Important follow-up diagnostic and other test results	see table and grafic
	10c	Intervention adherence and tolerability (How was this assessed?)	93+ff
	10d	Adverse and unanticipated events	none
Discussion	11a	A scientific discussion of the strengths AND limitations associated with this case report	139+ff
	11b	Discussion of the relevant medical literature with references.	105+ff
	11c	The scientific rationale for any conclusions (including assessment of possible causes)	134+ff
	11d	The primary "take-away" lessons of this case report (without references) in a one paragraph conclusion	140+f
Patient Perspective	12	The patient should share their perspective in one to two paragraphs on the treatment(s) they received	100+ff
Informed Consent	13	Did the patient give informed consent? Please provide if requested	Yes ☒ No ☐

Figure S1. CARE Checklist: For the original draft of the Case Report the CARE Cecklist was used to structure it.

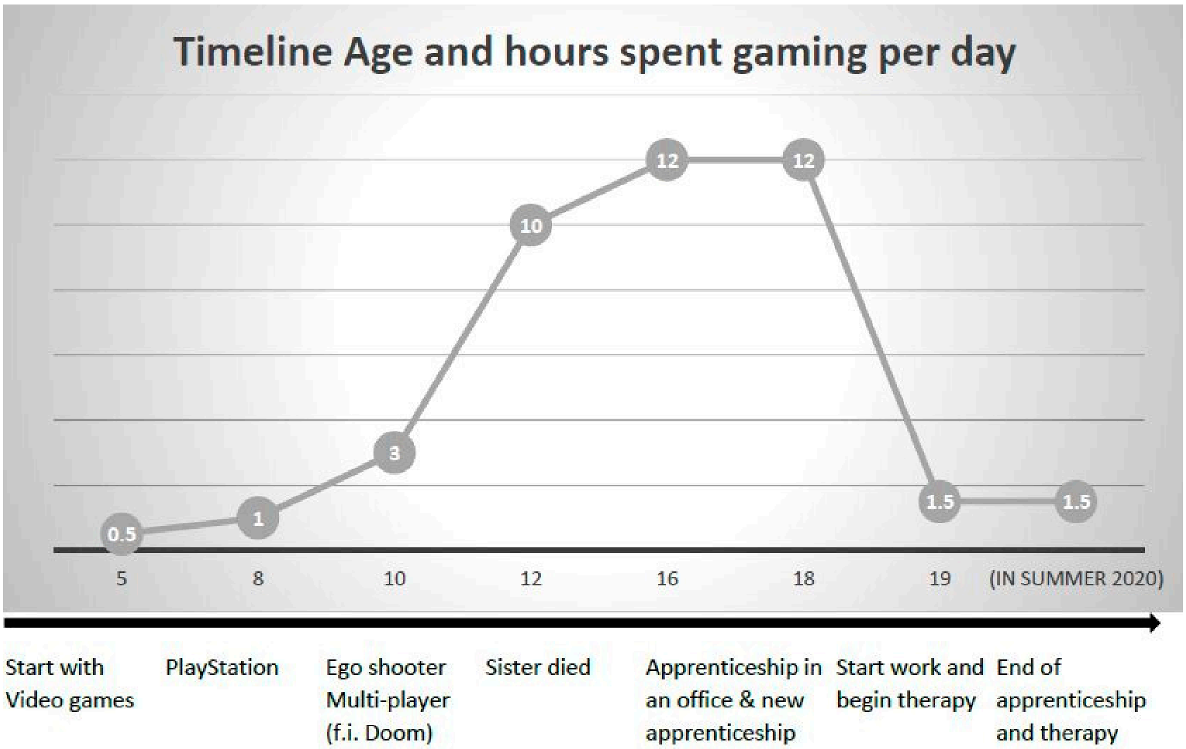

Figure S2. Timeline.
